# Structural and histone binding ability characterization of the ARB2 domain of a histone deacetylase Hda1 from *Saccharomyces cerevisiae*

**DOI:** 10.1038/srep33905

**Published:** 2016-09-26

**Authors:** Hui Shen, Yuwei Zhu, Chongyuan Wang, Hui Yan, Maikun Teng, Xu Li

**Affiliations:** 1 Hefei National Laboratory for Physical Sciences at Microscale, Innovation Center for Cell Signaling Network, School of Life Science, University of Science and Technology of China, Hefei, Anhui, 230026, People’s Republic of China; 2 Key Laboratory of Structural Biology, Hefei Science Center of CAS, Chinese Academy of Science, Hefei, Anhui, 230026, People’s Republic of China

## Abstract

Hda1 is the catalytic core component of the H2B- and H3- specific histone deacetylase (HDAC) complex from *Saccharomyces cerevisiae*, which is involved in the epigenetic repression and plays a crucial role in transcriptional regulation and developmental events. Though the N-terminal catalytic HDAC domain of Hda1 is well characterized, the function of the C-terminal ARB2 domain remains unknown. In this study, we determine the crystal structure of the ARB2 domain from *S. cerevisiae* Hda1 at a resolution of 2.7 Å. The ARB2 domain displays an α/β sandwich architecture with an arm protruding outside. Two ARB2 domain molecules form a compact homo-dimer via the arm elements, and assemble as an inverse “V” shape. The pull-down and ITC results reveal that the ARB2 domain possesses the histone binding ability, recognizing both the H2A-H2B dimer and H3-H4 tetramer. Perturbation of the dimer interface abolishes the histone binding ability of the ARB2 domain, indicating that the unique dimer architecture of the ARB2 domain coincides with the function for anchoring to histone. Collectively, our data report the first structure of the ARB2 domain and disclose its histone binding ability, which is of benefit for understanding the deacetylation reaction catalyzed by the class II Hda1 HDAC complex.

In eukaryotes, the structural unit of chromatin is the nucleosome that contains 147 bp of DNA wrapping around an octamer composed of two molecules each of the four histones-H2A, H2B, H3, and H4. The tails of these histones are unstructured and protruding from the core component^1^. Modifications could occur by the post-translational addition of small chemical compounds to the tails of the histones^2^, thus alter the properties of nucleosome and influence lots of fundamental biological processes. There are at least seven types of modifications identified on histones, including acetylation, methylation, phosphorylation, ubiquitylation, sumoylation, ADP ribosylation and deimination^3^,^4^,^5^,^6^,^7^.

Acetylation is one of the first discovered histone modification^8^, which occurs by the addition of acetyl group to the ε-amino of lysines in the N-terminal tail of histones. Histone lysine acetylation is highly reversible *in vivo*, and contains an abundant source of epigenetic information^9^. This modification is catalyzed by histone acetyltransferase enzymes (HATs) and reversed by histone deacetylases (HDACs)^10^. In eukaryotes, there are more than 10 different HDACs that can be grouped into two families (Rpd3/Hda1 family and sirtuin family) according to the ligands they need. HDACs can also be categorized into four classes according to phylogenetic analysis and sequence homology^11^,^12^. Rpd3/Hda1 family is zinc-dependent that contains class I, class II and class IV, with class II being further divided into two subclasses (IIa and IIb). Rpd3/Hda1 family is also designed as classical family so as to distinguish with the later found sirtuin family^13^. Sirtuin family composed of class III is NAD^+^-dependent^14^. The deacetylation mechanism of classical family is somewhat similar to the serine proteases. A bound water molecule is activated by both the zinc cation and a His-Asp charged relay system to attack the amide bond. The intermediate stabilized by the zinc cation and an adjacent tyrosine breaks up into acetate and lysine that accepts a proton from an activated histidine residue^15^.

Hda1 is a typical class II histone deacetylase from *Saccharomyces cerevisiae*. Two related proteins Hda2 and Hda3 are essential for the activity of Hda1 both *in vivo* and *in vitro*^16^,^17^. Together, they form the yeast Hda1 HDAC complex, which specifically deacetylates H2B and H3, with Hda1 homo-dimer as the catalytic subunit and Hda2-Hda3 hetero-dimer as the non-catalytic subunit. The details of interaction and domain organization of these three subunits have been well elaborated^18^. Hda2 and Hda3 both have an N-terminal DNA-binding domain (DBD) and a C-terminal coil-coil domain (CCD) that is used for Hda2 and Hda3 heterodimerization. The DBD domains of Hda2 and Hda3 share high sequence similarity and show structural homology to the C-terminal helicase lobes of SWI2/SNF2 chromatin-remodeling domains of the Rad54 family enzymes. Hda1 contains an N-terminal catalytic HDAC domain and a C-terminal ARB2 domain. The ARB2 domain is defined by sequence homology to the Arb2 protein, which is a subunit of the yeast Argonaute complex and is essential for histone H3 Lys9 (H3-K9) methylation, heterochromatin assembly, and siRNA generation^19^. However, until now, there are still no structural and functional indications for the ARB2 domain.

Here, we report the first structure of the ARB2 domain from *S. cerevisiae* Hda1. The ARB2 domain indicates structural resemblance to the α/β fold hydrolases. Largely distinct from these enzymes, the insertion region of the ARB2 domain protrudes from the core α/β fold and mediates the homodimer formation, which disrupts the corresponding substrate binding pocket. However, the unique homodimer architecture enables the ARB2 domain to bind to the histones. The ITC experiment shows that the ARB2 domain binds to the H2A-H2B dimer and H3-H4 tetramer with a *K*_d_ of 5.46 ± 0.69 μM and 3.24 ± 0.49 μM, respectively. Given the sequence homology, our results also provide some hints for understanding the function of the Arb2 protein, an essential subunit of the yeast Argonaute complex.

## Results

### Overall structure of the ARB2 domain of Hda1

**Figure 1:**
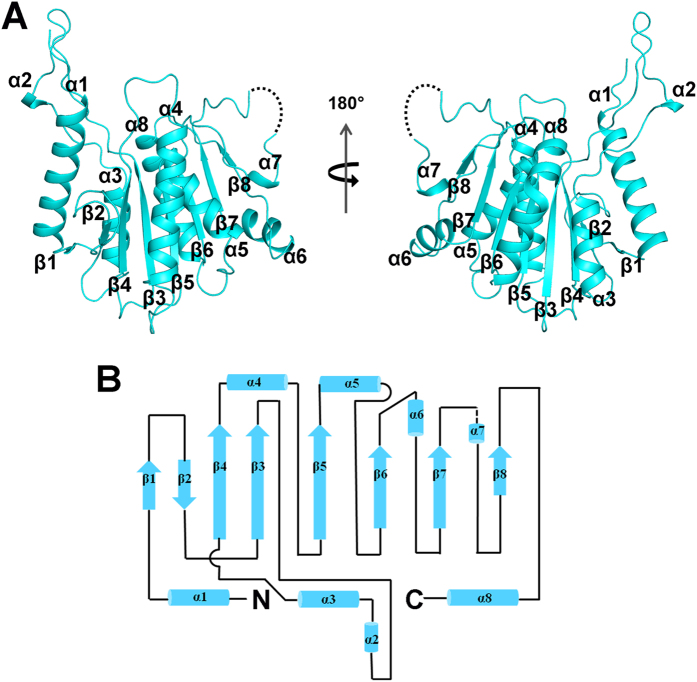
Overall structure of the ARB2 domain of Hda1. (**A**) Two views of the ARB2 domain are shown as a cartoon. α-helices and β strands are labelled and colored in cyan. Residues 659–663 are not observed and are shown as a dashed line. (**B**) Topological view of the structure of the ARB2 domain. Helices and strands are shown as cylinders and arrows, respectively.

Hda1 consists of an N-terminal catalytic domain and a C-terminal non-catalytic domain (ARB2). The catalytic domain of Hda1 shows high sequence homology to the HDACs of which the structures were reported and the catalytic mechanism has been well elucidated. However, the non-catalytic ARB2 domain is poorly defined. To gain insight into the role of the C-terminal non-catalytic domain of Hda1 functioning in the deacetylation process, we determined the crystal structure of ARB2 domain of Hda1. The structure was solved by the SAD method using the Se anomalous signal. The final model has been refined to 2.7 Å resolution and the details of the data collection and refinement statistics are summarized in Table 1. There is one ARB2 domain molecule observed in the crystal asymmetric unit. Due to the insufficient electron density, the residues 659–663 could not be traced. The ARB2 domain adopts an α/β sandwich architecture with an arm protruding outside (Fig. 1A). The core structure comprises an eight-stranded β-sheet (β1↑-β2↓-β4↑-β3↑-β5↑-β6↑-β7↑-β8↑), flanked by three α-helices on the proximal side (α1, α3 and α8) and four α-helices on the distal side (α4, α5, α6 and α7) (Fig. 1A,B). The protruded arm region spans residues 509–532, and contains a short α-helix (α2), (Fig. 1A).

### The homodimer interface of the ARB2 domian of Hda1

**Figure 2:**
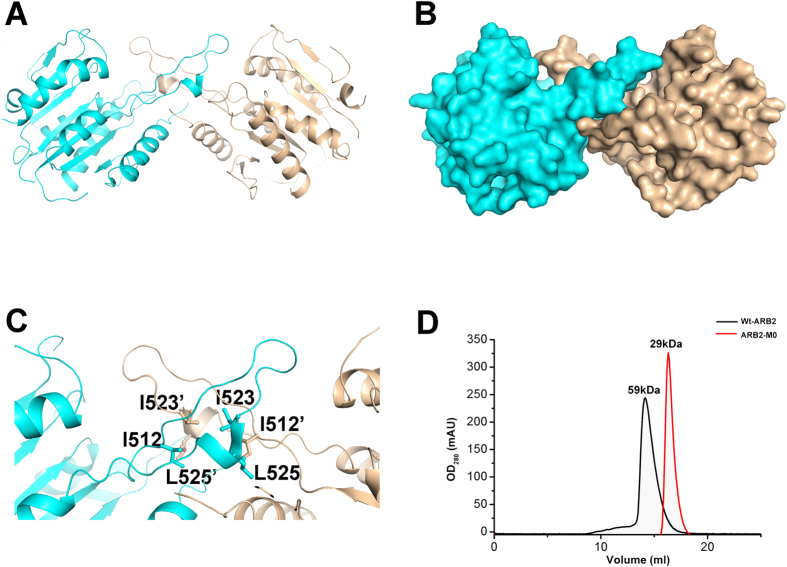
Dimerization of the ARB2 domain of Hda1. (**A**) Cartoon representation of the dimerization of the ARB2 domain. One molecule is colored in cyan, and the other one is colored in orange. (**B**) Surface show of the dimerization of the ARB2 domain. (**C**) The dimerization interface of the ARB2 domain. The residues involved in the dimerization interaction are labelled and shown as stick. (**D**) Gel-filtration analysis of the wild-type ARB2 domain and ARB2-M0.

**Figure 3:**
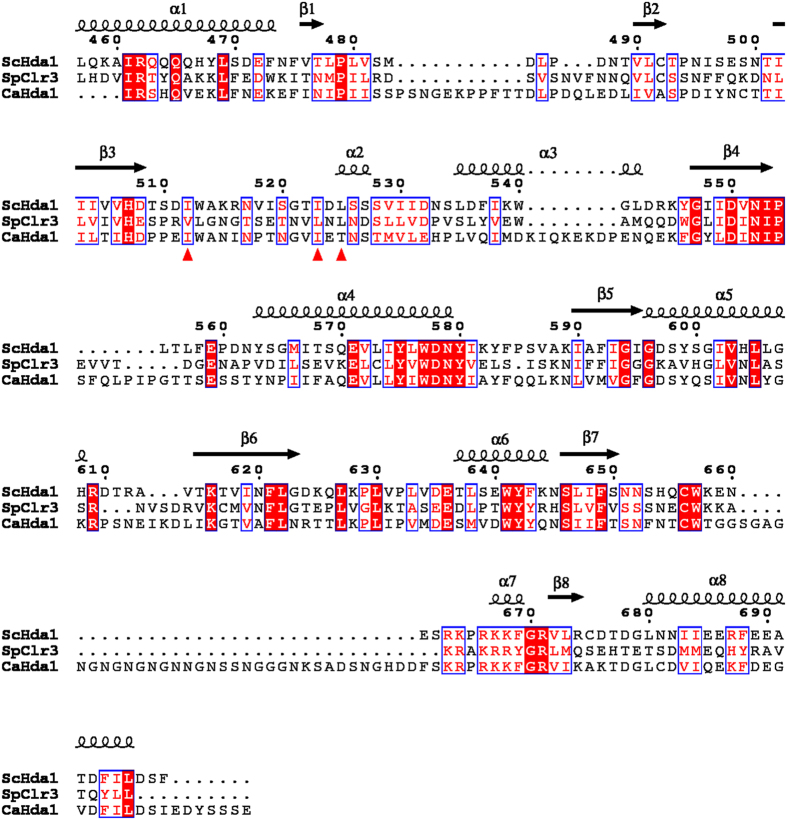
Structure-based sequence alignment of the ARB2 domain of *Saccharomyces cerevisiae* Hda1, *Schizosaccharomyces pombe* Clr3, and *Candida albicans* Hda1. Secondary structural elements are shown above the sequence. The key residues used for the dimer formation are marked by the red triangle.

Further examination of symmetry-related molecules indicates that two ARB2 domain molecules form a homodimer. As shown in Fig. 2A,B two symmetry-related ARB2 domain molecules come together to form an inverse “V” shape via the respective arm region. Residues Ile^512^, Ile^523^ and Leu^525^ located in the protruded arm region of each molecules form a hydrophobic core, which dominates the domain-domain interaction (Fig. 2C). The homodimer interface of these two ARB2 domain molecules buries 846.5 Å^2^ calculated by the PDBe PISA (Protein Interactions, Surfaces, Assemblies) server and scores 1.0 in Complex Formation Significance Score (CSS, ranges from 0 to 1 as interface relevance to complex formation increases)^20^. To confirm our structural observation and investigate the oligomer state of the ARB2 domain in solution, we performed the size-exclusion chromatography assay. As shown in Fig. 2D, the ARB2 domain eluted with a molecular weight of approximately 59 kDa, which is very close to the theoretical value of 58 kDa for the homodimer. Next, to investigate whether the hydrophobic core constituted by residues Ile^512^, Ile^523^ and Leu^525^ located in the arm region of each molecules mediates the homodimer interaction, we designed a ARB2 mutant, ARB2-M0 (residues Ile^512^, Ile^523^ and Leu^525^ mutated to Ala) to disrupt the homodimer interface. The size-exclusion chromatography assay shows that the mutant ARB2-M0 eluted with a molecular weight of approximately 29 kDa, which is coincident with the theoretical value of a monomer. Taken together, these results strongly suggest that the ARB2 domain of Hda1 exists as a homodimer, and this is consistent with the fact that the Hda1 protein functions as a dimer during the deacetylation reaction^17^. In addition, a structure-based sequence alignment shows that the protruded arm regions are conserved among the Hda1 homologies, and residues Ile^512^, Ile^523^ and Leu^525^ that play key role in the domain-domain interaction are relatively conserved (Fig. 3). We speculate that the ARB2 domain of these proteins may share a similar structural feature, containing a protruded arm region that mediates the homodimer formation.

### The ARB2 domain of Hda1 shows structural similarity to the α/β fold hydrolases

Considering that no structures exhibiting sequence homology to the ARB2 domain of Hda1 were deposited into the PDB database, the Dali server was used to search for the structures that are similar to the ARB2 domain. The result shows that the ARB2 domain is structural homologous to some hydrolases, such as the cinnamoyl esterase LJ0536 from *Lactobacillus johnsonii* (PDB code 3S2Z)^21^, with a Dali Z-score of 13.4 and an RMSD of 3.3 Å for 167 Cα atoms (Supplementary Figure 1A), the dienelactone hydrolase (YP_324580.1) from *Anabaena variabilis* ATCC 29413 (PDB code 2O2G), with a Dali Z-score of 12.9 and an RMSD of 3.4 Å for 166 Cα atoms (Supplementary Figure 1B), and the methyl dl-beta-acetylthioisobutyrate (dl-MATI) esterase from *Pseudomonas putida* IFO 12996 (PDB code 1ZOI)^22^, with a Dali Z-score of 12.7 and an RMSD of 3.0 Å for 151 Cα atoms (Supplementary Figure 1C). Even though these structures share a similar core architecture containing a central eight-stranded β-sheet flanked by α-helices on each side, there are still obvious structural variations in the inserted subdomains of each protein. Largely distinct from these enzymes, the insertion region of the ARB2 domain protrudes from the core α/β fold, which functions to mediate the homodimer formation instead of constituting a substrate binding pocket. Thus, the biological role of the ARB2 domain of Hda1 in the histone deacetylation reaction is an interesting question that deserves further exploration.

### The ARB2 domain of Hda1 possesses the histone binding ability

**Figure 4:**
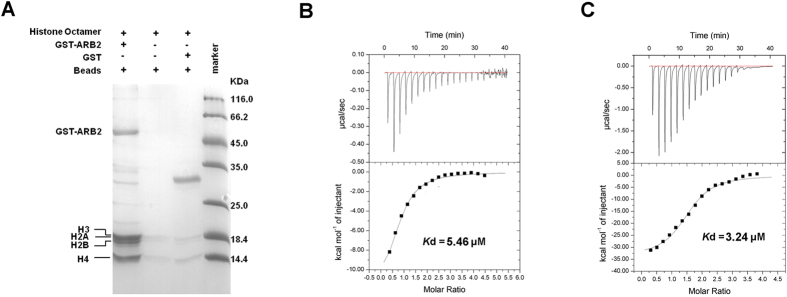
Histones binding property of the ARB2 domain of Hda1. (**A**) Pull-down assay of the ARB2 domain with histone octamer. The bands corresponding to H3, H2A, H2B and H4 are indicated by the arrows. ITC profile of the ARB2 domain titrated against H2A-H2B dimer (**B**) and H3-H4 tetramer (**C**).

Given that the Hda1 protein is the catalytic subunit of yeast class II Hda1 HDAC complex, and is directly involved in the histone deacetylation reaction, we wonder whether the ARB2 domain of Hda1 possesses the histone binding ability. We reconstituted the yeast histone octamer *in vitro*, and performed the GST pull-down assay to investigate the interaction between the histone octamer and the ARB2 domain. As shown in Fig. 4A, the ARB2 domain exhibited a prominent binding affinity with the histone octamer. Then, we performed the ITC experiment to quantitatively investigate the histone-binding preference of the ARB2 domain. We reconstituted the yeast histone H2A-H2B dimer and H3-H4 tetramer *in vitro*. The ITC experiment shows that the ARB2 domain could bind to both the H2A-H2B dimer and H3-H4 tetramer, with a *K*_d_ of 5.46 ± 0.69 μM and 3.24 ± 0.49 μM, respectively (Fig. 4B,C). These results could well support our hypothesis, and provide the first functional characterization of the ARB2 domain.

### Probing the residues involved for accommodating the histones

**Figure 5:**
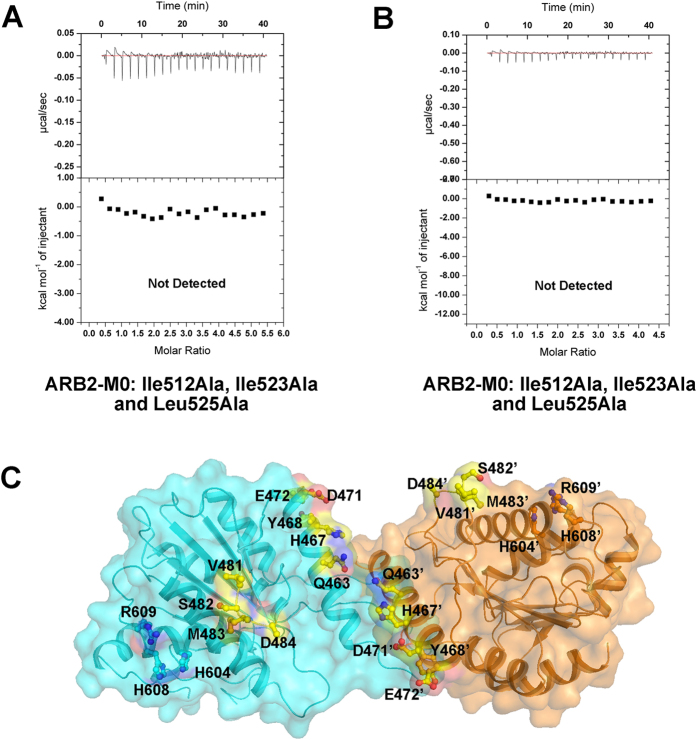
Dimerizaiton of the ARB2 domain is essential for histones binding. ITC profile of the mutant ARB2-M0 titrated against H2A-H2B dimer (**A**) or H3-H4 tetramer (**B**). (**C**) Two ARB2 domain molecules come together to form an inverse “V” shape. The residues located in the groove of the “V” shape are labelled and shown as stick.

**Figure 6:**
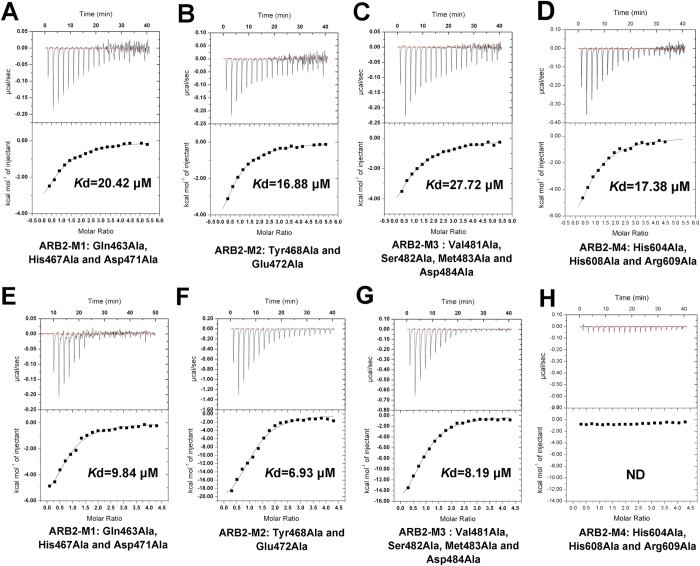
ITC profile of the ARB2 domain mutants titrated against H2A-H2B dimer and H3-H4 tetramer. (**A**) The mutant ARB2-M1 titrated against H2A-H2B dimer. (**B**) The mutant ARB2-M2 titrated against H2A-H2B dimer. (**C**) The mutant ARB2-M3 titrated against H2A-H2B dimer. (**D**) The mutant ARB2-M4 titrated against H2A-H2B dimer. (**E**) The mutant ARB2-M1 titrated against H3-H4 tetramer. (**F**) The mutant ARB2-M2 titrated against H3-H4 tetramer. (**G**) The mutant ARB2-M3 titrated against H3-H4 tetramer. (**H**) The mutant ARB2-M4 titrated against H3-H4 tetramer.

As discussed previously, two ARB2 domain molecules form an inverse “V” shape via the protruded arm region. To investigate whether the dimer architecture of the ARB2 domain is essential for interacting with the histones, we performed the ITC experiment to measure the binding affinity of ARB2-M0 to H2A-H2B dimer and H3-H4 tetramer, respectively. As shown in Fig. 5A,B, the mutant ARB2-M0 completely destroyed the interaction with histones, indicating the vital role of the dimer architecture for histones binding. Next, we investigate whether the groove enclosed by two ARB2 domain molecules is used for accommodating the histones. We mutated some residues surrounded the groove and constructed four mutants, ARB2-M1 (residues Gln^463^, His^467^ and Asp^471^ mutated to Ala), ARB2-M2 (residues Tyr^468^ and Glu^472^ mutated to Ala), ARB2-M3 (residues Val^481^, Ser^482^, Met^483^ and Asp^484^ mutated to Ala) and ARB2-M4 (residues His^604^, His^608^ and Arg^609^ mutated to Ala) (Fig. 5C). Next, we performed the ITC experiment to measure the binding affinity of these mutants to H2A-H2B dimer and H3-H4 tetramer, respectively. As shown in Fig. 6 and Table 2, the binding affinity of mutants ARB2-M1, ARB2-M2, ARB2-M3 and ARB2-M4 to H2A-H2B dimer is 20.42 ± 2.77 μM, 16.88 ± 1.45 μM, 27.72 ± 3.33 μM and 17.38 ± 2.72 μM, respectively. Correspondingly, the binding affinity of mutants ARB2-M1, ARB2-M2 and ARB2-M3 to H3-H4 tetramer is 9.84 ± 1.48 μM, 6.93 ± 1.24 μM, and 8.19 ± 0.72 μM, respectively. The mutant ARB2-M4 completely loses the binding ability to H3-H4. The ITC results show that all of these mutants could weaken the interaction between the ARB2 domain and histones, suggesting that the inverse “V” shape groove is indeed involved in interacting with histones and the residues His^604^, His^608^ and Arg^609^ are indispensable for binding to H3-H4.

## Discussion

The yeast class II Hda1 HDAC complex shows similarity to the mammalian class II HDACs, such as HDAC4, HDAC5, HDAC6, HDAC7, HDAC9 and HDAC10, which play an essential role in nucleosomal repression of transcription. In *Saccharomyces cerevisiae*, the Hda1 HDAC complex is recruited to specific promoters through the transcriptional corepressor Tup1. The N-terminal repressor domain of Tup1 could make direct interaction with the catalytic subdomain of the Hda1 HDAC complex^23^. Besides, a Tup1-independent genome-wide non-specific transcriptional repression by the Hda1 HDAC complex is also prevalent in *Saccharomyces cerevisiae*^24^. It was reported that more than 69% sites deacetylated by the Hda1 HDAC complex are not affected by a *TUP1* knock-out mutation^25^. Further studies disclosed that both the N-terminal DBD domains of the two subunits Hda2 and Hda3 of the Hda1 HDAC complex possess the non-specific DNA-binding ability, which may serve as an anchor to hold the catalytic subunit Hda1 for deacetylation. Although the catalytic domain of Hda1 has been well elucidated, the non-catalytic ARB2 domain is poorly defined. To gain insight into the role of the C-terminal non-catalytic domain of Hda1, we determine the structure of the ARB2 domain of Hda1. The ARB2 domain adopts an α/β sandwich fold, and two symmetry-related ARB2 domain molecules come together to form an inverse “V” shape through the protruding arm. This is consistent with previous studies that the Hda1 protein must form a homodimer to execute the histone deacetylase activity both *in vivo* and *in vitro*^17^. Unexpectedly, the ARB2 domain could make direct interaction with the yeast core histones and the dimer architecture is essential for the interaction. Additionally, the groove enclosed by two ARB2 molecules is involved in histone binding.

As we known, histone octamer is wrapped by DNA to form nucleosome in chromosome and the physical character of the histone octamer is totally different from that of the nucleosome. Although our results indicate that the ARB2 domain of Hda1 can bind to histone octamer, it is important to investigate how the ARB2 domain functions *in vivo*. That may come from the multi-component of the yeast Hda1 HDAC complex and unique features to fulfill the function. As reported, the histone deacetylase Hda1 could not exert the histone deacetylase activity alone, and two related proteins Hda2 and Hda3 are essential for the activity of Hda1. Besides, posttranslational modifications of core histone proteins are usually accompanied with chromosome remodeling. Generally, acetylated histones are correlated with more open chromatin and active gene expression, whereas deacetylated histones are correlated with closed chromatin and repressed gene expression. The Hda1 homologue in fission yeast (*S. pombe*), Clr3, forms a multi-protein complex *in vivo*, which recruits helicase Mit1 to perform both deacetylation and chromatin-remodeling functions^26^. We propose that the yeast class II Hda1 HDAC complex may work in a similar way, and it coincides with the report that the non-catalytic subunits Hda2 and Hda3 share high sequence similarity and show structural homology to the C-terminal helicase lobes of SWI2/SNF2 chromatin-remodeling domains of the Rad54 family enzymes. Therefore, we speculate that the histone binding ability of the ARB2 domain of Hda1, to a large extent, may function as a histone chaperone and be involved in the chromatin remodeling process.

The ARB2 domain of Hda1 is the first structural and functional characterized member of the Arb2 family. Given the sequence homology similarity, we speculate that the Arb2 protein, to a large extent, possesses the histone binding ability. This will greatly facilitate us to understand how the Argonaute complex works in the progress of histone methylation, heterochromatin assembly, and siRNA generation. In addition, whether the ARB2 domain existed as a conserved histone binding module in other members of the Arb2 family is an interesting question that deserves to address. Further experiments are in progress to confirm this hypothesis.

## Methods

### Cloning, overexpression and purification

The gene encoding the C-terminal ARB2 domain of Hda1 (Hda1^457–698^) was amplified from *Saccharomyces cerevisiae* genome, and cloned into modified pET-28a vector (Novagon) with six-His tag at the N-terminus and pGEX-4T-2 vector (Novagon). The mutations of the ARB2 domain were generated using the MutanBEST Kit (Takara). Overexpression of all recombinant proteins was induced in *Escherichia coli* BL21 (DE3) cells (Novagen) with 0.5 mM IPTG when the cell density reached an OD_600 nm_ of 0.6–0.8. After growth for approximately 20 h at 16 °C, the cells were collected and lysed. The recombinant proteins were purified using Ni^2+^ -nitrilotriacetate affinity resin (Ni–NTA; Qiagen) in buffer (25 mMTris–HCl pH 7.5, 200 mM NaCl) and eluted with 500 mM imidazole. The proteins were further purified using HiTrap Q FF (5 ml) and HiLoad 16/60 Superdex 200 (GE Healthcare). The final proteins were concentrated to 15 mg/ml for crystallization trials. Since there were no homologous structures published, a selenomethionine derivative Hda1^457–698^ was obtained to solve the phase problem. The selenomethionine derivative Hda1^457–698^ was overexpressed in the same competent cells as native Hda1^457–698^ but using M9 medium based on a methionine-biosynthesis inhibition method. The purification of selenomethionine derivative Hda1^457–698^ followed the same protocol as used for the native Hda1^457–698^.

The ORF of yeast histones H2A, H2B, H3 and H4 were amplified from *Saccharomyces cerevisiae* genome, and cloned into pET-22b (Novagon) vector with six-His tag at the C terminus. The recombinant plasmids were transformed into the *E. coli* Rosseta (DE3) strain for expression. The yeast reconstituted histones were prepared as described previously^27^,^28^.

### Crystallization, data collection and structure determination

Crystals of both the native Hda1^457–698^ and selenomethionine derivative Hda1^457–698^ were grown using the hanging-drop vapour diffusion method at 285 K and grew to maximum size in approximately 1 day in the buffer containing 0.1 M Sodium acetate trihydrate pH 4.6, 2.0 M Sodium formate. For data collection, crystals were transferred to cryoprotectant solution consisting of the respective reservoir solution supplemented with 25% (v/v) glycerol and then flash-cooled in liquid nitrogen. Data sets for all crystals were collected at 100 K on beamline BL17U of synchrotron-radiation at Shanghai Synchrotron Radiation Facility (SSRF). The data sets were processed and scaled with *HKL*-2000 and programs from the *CCP*4 package^29^. The structure of Se-Hda1^457–698^ was determined by the single-wavelength anomalous dispersion (SAD) phasing technique using *AutoSol* as implemented in *PHENIX*^30^. The initial model was built automatically using *AutoBuild* in *PHENIX.* Using the Se- Hda1^457–698^ structure as the search model, the structure of native Hda1^457–698^ was determined by the molecular-replacement method using *MOLREP*^31^ as implemented in *CCP4i*. All of the initial models were refined using the maximum-likelihood method implemented in *REFMAC5*^32^ as part of the *CCP4* program suite and rebuilt interactively using *Coot*^33^. The final models were evaluated with *MolProbity*^30^ and *PROCHECK*^34^. The crystallographic parameters are listed in Table 1. All of the figures showing structures were prepared with *PyMOL.*

### Size-exclusion chromatography assay

The apparent molecular masses of the ARB2 domain of Hda1 and the ARB2-M0 mutant were estimated with a Superdex 200 column (10/300 GL; GE Healthcare). Briefly, protein samples or molecular-mass standards were applied onto the Superdex 200 column at a flow rate of 0.5 ml/min with 25 mM Tris-HCl pH 7.5, 200 mM NaCl. The standard proteins (GE Healthcare) used in this assay were β-amylase (200.0 kDa), alcohol dehydrogenase (150.0 kDa), albumin (66.0 kDa), carbonic anhydrase (29.0 kDa) and cytochromec(12.4 kDa). The void volume was determined with blue dextran (GE Healthcare).

### Isothermal titration calorimetry (ITC) experiments

The ITC binding studies were performed using an ITC200 (GE) at 20 °C with 0.04 ml of 0.5 mM ARB2 or ARB2 mutants (M0, M1, M2, M3, M4) in the injector cell and 0.3 ml of 0.2 mM H2A/B or 0.25 mM H3/4 in the sample cell, respectively. All proteins were kept in a buffer consisting of 25 mM Tris-HCl pH 7.5 and 200 mM NaCl. Twenty microliters injection volumes were used for all experiments. Two consecutive injections were separated by 2 min to reset the baseline. The control experiment, consisting of titration of ARB2 against buffer, was performed and subtracted from each experiment. ITC data was analyzed with a single-site fitting model, using Origin 8.6 (OriginLab Corp).

### GST pull-down assay

A similar amount of purified recombinant GST-ARB2 and GST were immobilized on 100 μl of GST resin. After washing twice (1 ml each time) with buffer C, the beads were incubated with histone octamer for 1 h. Then, the beads were washed thoroughly with buffer C. Finally the beads were mixed with 100 μl of 2 × SDS-PAGE loading buffer and boiled for 10 min at 90 °C. The samples were stained with Coomassie Brilliant Blue.

## Additional Information

**How to cite this article**: Shen, H. *et al*. Structural and histone binding ability characterization of the ARB2 domain of a histone deacetylase Hda1 from *Saccharomyces cerevisiae. Sci. Rep.* **6**, 33905; doi: 10.1038/srep33905 (2016).

## Supplementary Material

Supplementary Information

## Figures and Tables

**Table 1:** Data collection and refinement statistics of the Hda1^457–698^ (ARB2 domain).

	Se-Hda1^457–698^	Native-Hda1^457–698^
Data collection		
Space Group	*P*3_1_21	*P*3_1_21
Unit Cell Parameters		
*a, b, c* (Å)	106.1, 106.1, 89.0	105.0, 105.0, 87.0
*α, β, γ* (˚)	90.0, 90.0, 120.0	90.0, 90.0, 120.0
Wavelength(Å)	0.9792	0.9792
Resolution limits(Å) a	50.00–3.10(3.21–3.10)	50.00–2.70(2.75–2.70)
No. of unique reflections	10771	14972
Completeness (%)	99.2(100.0)	98.1(96.9)
Redundancy	19.4(20.0)	10.0(10.3)
*R*_merge_ (%) b	9.2(77.1)	8.6(67.2)
Mean I/σ(I)	32.9(5.0)	14.7(3.1)
Refinement		
Resolution limits(Å)		50.00–2.70
*R*_work_(%) c/*R*_free_(%) d		18.98/23.82
Rmsd for bonds (Å)		0.009
Rmsd for angles (˚)		1.281
B factor (Å^2^)		81.4
No. of non-hydrogen protein atoms		1933
Ramachandran plot (%)		
most favored regions		92.0
additional allowed regions		8.0
PDB code		5J8J

^a^Values in parentheses are for the highest-resolution shell.

^b^*R*_*merge*_ = ∑|I_i_ − <I>|/∑|I|, where I_i_ is the intensity of an individual reflection and <I> is the average intensity of that reflection.

^c^*R*_*work*_ = ∑||F_o_| − |F_c_||/∑|F_o_|, where F_o_ and F_c_ are the observed and calculated structure factors for reflections, respectively.

^d^*R*_*free*_ was calculated as *R*_*work*_ using the 5% of reflections that were selected randomly and omitted from refinement.

**Table 2:** The thermodynamic parameters of the ITC experiments.

	Proteins	∆H kcal/mol	∆S cal/mol/deg	*K*_D_ μM	N
H2A/B	ARB2	−12.88	−19.8	5.46 ± 0.69	0.78
	M0			ND	
	M1	−6.86	−1.90	20.42 ± 2.77	0.77
	M2	−8.90	−8.50	16.88 ± 1.45	0.64
	M3	−11.55	−18.50	27.72 ± 3.33	0.74
	M4	−11.46	−17.3	17.38 ± 2.72	0.80
H3/4	ARB2	−33.85	−90.30	3.24 ± 0.49	1.52
	M0			ND	
	M1	−7.63	−3.07	9.84 ± 1.48	0.78
	M2	−24.12	−58.60	6.93 ± 1.24	1.10
	M3	−20.19	−45.60	8.19 ± 0.72	0.90
	M4			ND	
