# Supplementary material for: Structural and histone binding ability characterization of the ARB2 domain of a histone deacetylase Hda1 from Saccharomyces cerevisiae
Source: Sci Rep. 2016 Sep 26;6:33905. doi: 10.1038/srep33905 (PMC5036196; doi:10.1038/srep33905)
Supplement: Supplementary Information [file srep33905-s1.pdf]

Structural and histone binding ability characterization of the ARB2 domain of a histone deacetylase Hda1 from *Saccharomyces cerevisiae*

Hui Shen[1,2,+], Yuwei Zhu[1,2,+], Chongyuan Wang[1,2], Hui Yan[1,2], Maikun Teng[1,2]* and Xu Li[1,2]*

[1] Hefei National Laboratory for Physical Sciences at Microscale, Innovation Center for Cell Signaling Network, School of Life Science, University of Science and Technology of China, Hefei, Anhui, 230026, People's Republic of China

[2] Key Laboratory of Structural Biology, Hefei Science Center of CAS, Chinese Academy of Science, Hefei, Anhui, 230026, People's Republic of China

* Correspondence should be addressed to X.L. (email: sachem@ustc.edu.cn) or M.T. (email: mkteng@ustc.edu.cn)

[+] These authors contributed equally to this work

Supplementary Figure 1

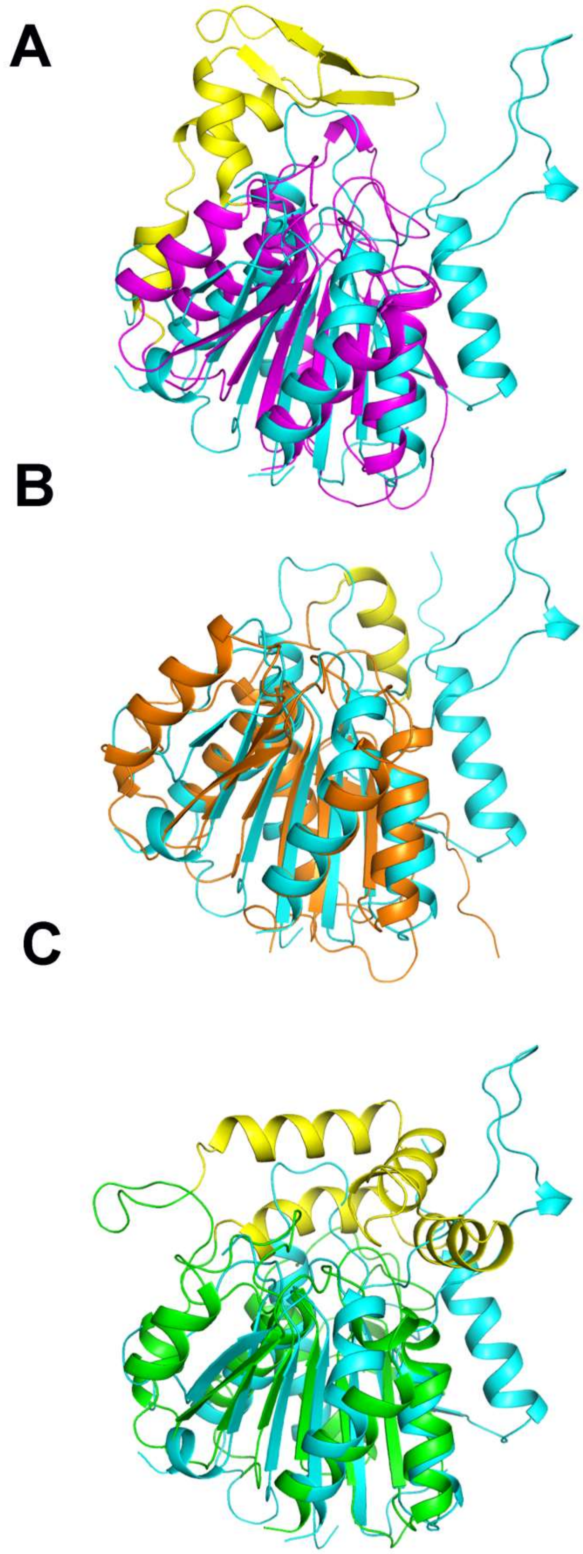

Supplementary Figure 1. Structural superposition of the ARB2 domain of Hda1 with cinnamoyl esterase LJ0536 from *Lactobacillus johnsonii* (A), the dienelactone hydrolase (YP_324580.1) from *Anabaena variabilis* ATCC 29413 (B) and the methyl dl-beta-acetylthioisobutyrate esterase from *Pseudomonas putida* IFO 12996 (C). The ARB2 domain of Hda1 are colored in cyan. The cinnamoyl esterase LJ0536, dienelactone hydrolase (YP_324580.1) and methyl dl-beta-acetylthioisobutyrate esterase are colored in magenta, orange and green, respectively. The inserted subdomain of the cinnamoyl esterase LJ0536, dienelactone hydrolase (YP_324580.1) and methyl dl-beta-acetylthioisobutyrate esterase are colored in yellow. The inserted subdomain of the cinnamoyl esterase LJ0536 displays an open canal-like feature, consisting of three α-helices and two short β-hairpins. It constitutes the unique substrate binding pocket of the cinnamoyl esterase and is essential for the enzymatic activity. Instead, the dienelactone hydrolase (YP_324580.1) just contains an α-helix insertion between β4 and helix α1, and the substrate recognition mechanism is still unreported. The methyl dl-beta-acetylthioisobutyrate (dl-MATI) esterase assembles as a homotrimer in both crystals and solution. Each monomer possesses a four helices insertion that encloses a cavity used for substrate accommodation.
